# The EORTC QLQ-F17 as a shortened version of the EORTC QLQ-C30 to assess self-reported functioning in cancer patients: investigating equivalence and psychometric properties in a randomized cross-over trial

**DOI:** 10.1016/j.eclinm.2025.103262

**Published:** 2025-06-03

**Authors:** Florian Zeman, Johannes M. Giesinger, Tobias Pukrop, Morten Aa Petersen, Mogens Groenvold, Sandra Nolte, Dagmara Kuliś, Sunil Shrestha, Laurence Leysen, Kim Cocks, Corneel Coens, Georgios Ioannidis, Cecilia Pompili, Michael Koller, of the EORTC QLG

**Affiliations:** a Center for Clinical Studies, University Hospital Regensburg, Regensburg, Germany; b Bavarian Cancer Research Center (BZKF), Regensburg, Germany; c Department of Psychiatry, Psychotherapy, Psychosomatics, and Medical Psychology, Innsbruck Medical University, Innsbruck, Austria; d Department of Internal Medicine III, Hematology and Oncology, University Hospital Regensburg, Regensburg, Germany; e Palliative Care Research Unit, Department of Geriatrics and Palliative Medicine GP, Bispebjerg and Frederiksberg Hospital, University of Copenhagen, Copenhagen, Denmark; f Department of Public Health, University of Copenhagen, Copenhagen, Denmark; g Person-Centred Research, Eastern Health Clinical School, Monash University, Melbourne, VIC, Australia; h School of Health Sciences, Swinburne University of Technology, Melbourne, VIC, Australia; i European Organisation for Research and Treatment of Cancer (EORTC), Quality of Life Department, Brussels, Belgium; j Department of Research and Academics, Kathmandu Cancer Center, Tathali, Bhaktapur, Nepal; k Person-Centred Research, Eastern Health Clinical School, Monash University, Melbourne, VIC, Australia; l Statistics and Programming, Adelphi Values, Bollington, Cheshire, England, UK; m Oncology Department, Nicosia General Hospital, State of Health Services Organisation, Nicosia, Cyprus; n University of Hull, Hull and York Medical School, Hull, UK

**Keywords:** Quality of life, Patient-reported outcomes, EORTC QLQ-C30, EORTC QLQ-F17, Equivalence, Psychometric properties

## Abstract

**Background:**

The EORTC QLQ-F17, a shortened version of the EORTC QLQ-C30, solely contains the items related to functioning and omits symptom scales. The QLQ-F17 is conceived an equivalent to the functional part of the QLQ-C30, but to date there is no empirical evidence to support this rationale.

**Methods:**

This randomized, cross-over, multi-national, questionnaire-based study investigates the equivalence and psychometric properties of the QLQ-F17 compared to the functional scales of the QLQ-C30. Respondents had to fill in both questionnaires, the order of which was presented in a randomized and balanced manner (QLQ-C30—QLQ-F17 vs. QLQ-F17—QLQ-C30). Equivalence testing used Differential Item Functioning (DIF) and linear models. The margin of equivalence was set at ]–5, 5[ points. The study is registered with ClinicalTrials.gov (NCT05479682). Patients were enrolled between February 17 and March 28, 2023.

**Findings:**

A total of 2672 cancer patients of all major cancer types, aged between 18 and 92 years with an equal gender distribution (50/50) from 11 countries, were recruited between 17 February and 28 March 2023. Adjusted mean differences between QLQ-C30 and QLQ-F17 ranged between −1·55 and 3·25 for all scales, and the limits of the 95%-CIs, ranging from −3·11 to 4·87, were all within the equivalence margin. All effect sizes of the DIF analyses were <0·01. For the scales of the QLQ-F17, Cronbach's Alpha ranged from 0·73 to 0·89, item–own scale correlations from 0·47 to 0·78 and item–other scale correlations from 0·19 to 0·70.

**Interpretation:**

The QLQ-F17 yielded score values that are equivalent to the functional part of the QLQ-C30. Consequently, future clinical studies can employ the QLQ-F17 as a generic tool without losing comparability to studies using the QLQ-C30. Supplementing the QLQ-F17 with relevant symptom items from the EORTC Item Library allows for a time-efficient and flexible measurement strategy.

**Funding:**

This research was funded by the 10.13039/501100004897European Organisation for Research and Treatment of Cancer (EORTC) Quality of Life Group, grant number 010/2021.

**Research in context**

**Evidence before this study**

This study represents a pioneering investigation into the recently developed QLQ-F17 questionnaire. The QLQ-F17 was introduced in a conference proceeding, but to date, there exists no empirical literature, as demonstrated by a search for articles, grey literature, and clinical studies in PubMed, Embase, Web of Science Core Collection (SCI-EXPANDED, SSCI), Cochrane Library, ClinicalTrials.gov and Google Scholar. A broader PubMed search on “functioning scales” and “patient-reported outcomes” yielded only 22 articles, of which only one explored the equivalence of the functional scales with a more comprehensive long-form questionnaire.

**Added value of this study**

This study establishes the QLQ-F17 as an equivalent to the QLQ-C30 to assess functioning in cancer patients in terms of score values and psychometric properties. The QLQ-F17 is in line with the requirements of the FDA to provide short, focused, and psychometrically sound measures to assess functioning in cancer patients.

**Implications of all the available evidence**

The QLQ-F17 can now be considered in future research, ensuring comparability between studies employing the QLQ-C30. Somatic symptoms that are relevant in a given research context can be derived from the EORTC item library.

## Introduction

The European Organisation for Research and Treatment of Cancer (EORTC) developed a modular approach to quality of life (QOL) assessment for cancer clinical trials and daily practice, encompassing a generic core questionnaire (EORTC QLQ-C30) and a large range of modules referring to tumour- or treatment-specific symptoms.^1^ The QLQ-C30 has proven content validity,^2^ has been translated into over 130 language versions and is one of the most widely used questionnaires in the field. As the name suggests, it contains 30 items, of which 13 are related to symptoms (e.g., pain) and 17 to aspects of functioning, which refer to the ability of cancer patients to perform various activities and tasks in their daily lives.

Regulatory bodies and many users advocate for short questionnaires to reduce patient-burden while capturing all relevant information in a given clinical context. In response to this pragmatic request, the EORTC has prepared a shortened version of the QLQ-C30, the QLQ-F17, that includes solely the functional scales of the QLQ-C30. The QLQ-F17 can be supplemented by symptom-specific modules or an appropriate set of single items from the EORTC Item Library.^3^ This allows for a flexible, easily-implemented and time-efficient testing strategy while following the multimodality approach of modern cancer care.

The QLQ-F17 can be considered as a short equivalent to the functional part of the QLQ-C30, which would allow for comparability of studies that either use the QLQ-C30 or the QLQ-F17 as the generic form. This rationale, however, requires evidence that the two forms yield the same scores on respective functional scales. In other words, results of functional scales should be equivalent, no matter whether measured with the QLQ-C30 or the QLQ-F17.

Equivalence of the two forms cannot be taken for granted. Research on response biases and item order effects has shown that preceding questions can influence the responses to subsequent questions.^4^^,^^5^ When responding to a question, people tend to utilize prior information. They may integrate previous thoughts or considerations in the sense of consistency (i.e., response to question B will become consistent with response to question A) or in the sense of contrast (i.e., response to question B will become different from response to question A, in order to avoid redundancy). In any event, these phenomena may introduce measurement bias. It can be argued that the elimination of all the QLQ-C30 symptom, and financial difficulties items could alter the manner in which subsequent questions are answered. For instance, respondents who report high degrees of dyspnoea and pain in these scales might report low emotional functioning in comparison with respondents with the same (objective) health status who were not presented with items on these symptoms and thus were not made aware of their poor health. On the contrary, patients who report no symptoms on the symptom scales may infer that their health is excellent and consequently will report good functioning on the functional scales, more so than patients with equal objective health but who had not responded to the symptom scales. These effects need to be considered when an adapted/shortened questionnaire version is developed based on an original one.

Thus, the primary aim of the study was to investigate whether the QLQ-F17 and the QLQ-C30 are equivalent in the sense of yielding equivalent measurement results with regard to the scales they share in common (PF, RF, CF, EF, SF and QL), as well as whether basic psychometric properties of the scales such as internal consistency or scaling error are equivalent.

## Methods

### Study design

This study is a randomized, cross-over, multi-national questionnaire-based study investigating the equivalence of the functional scales of the QLQ-C30 and QLQ-F17.

Available translations of the questionnaire, developed according to the EORTC translation guidelines,^6^ were used for each language region. Approval was obtained from the Ethics Committee of the University of Regensburg, Regensburg, Germany (27th July 2022, reference number 22-3018-104). The study was registered at ClinicalTrials.gov (NCT05479682).

### Assessments

The QLQ-C30 assesses general aspects of quality of life of cancer patients. It includes five functional scales—physical (PF), role (RF), cognitive (CF), emotional (EF), and social (SF)—a Global Health Status/Quality of Life (QL) scale, three symptom scales—fatigue, pain, and nausea/vomiting—as well as six single items (dyspnoea, loss of appetite, insomnia, constipation, diarrhoea, and financial difficulties). All items are scored on a 4-point Likert scale ranging from 1 (not at all) to 4 (very much), except the two items of the QL-scale that are scored on a 7-point scale ranging from 1 (very poor) to 7 (excellent). In accordance with the QLQ-C30 scoring manual,^7^ sum scores of the scales were standardized by a linear transformation, resulting in scores ranging from 0 to 100. Higher scores in functional scales indicate a higher level of functioning; higher scores in symptom scales and in single items indicate a higher degree of impairment.

The QLQ-F17 is a shortened version of the QLQ-C30, in which all scales and single items on symptoms and financial difficulties have been omitted. Thus, the 17-item questionnaire includes just the five functional scales (PF, RF, CF, EF, and SF) and the QL-scale.

**Fig. 1:**
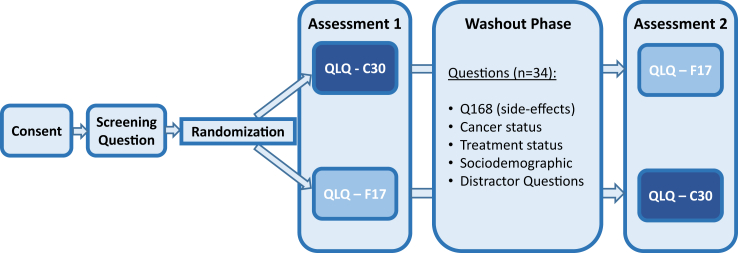
Study design.

The QOL questionnaires were presented in one single session using a cross-over design (Fig. 1), where patients were assigned to one of two groups i.e., QLQ-C30 first followed by the QLQ-F17 (C—F) vs. QLQ-F17 followed by the QLQ-C30 (F—C) using the least-fill-in algorithm, i.e., by allocating a newly enrolled patient to the group with the lowest number of survey participants at the given point in time. This approach emulates block randomization by ensuring an equal number of respondents in each group and maintaining structural equivalence between the groups to be compared. Allocation concealment was secured since survey providers were unaware of the characteristics of the respondents and allocation to one of the two groups was solely computer based. Additionally, allocation concealment was ensured by withholding information about the group assignments from the patients.

Between the administrations of the two questionnaires, patients had to work on an intermediate task consisting of 34 questions to weaken the memories of the first set of QoL items before the alternative QLQ was presented. Some questions addressed cancer-related health issues (type of tumour, time since diagnosis, active treatment, ECOG performance status) and sociodemographic characteristics (age, sex, level of education). Most items were distractor questions, tapping into various issues such as favourite food, travelling, or music.

### Sampling/data collection

Sampling was done by the panel research company Kantar (http://www.kantar.com) using an internet-based survey tool. Kantar is certified according to ISO 20252:2019, specializes in health studies, and maintains patient panels with various diagnoses, including cancer. We collected data via a digital survey and applied the EORTC Guidelines on the implementation and management of EORTC QOL Instruments in electronic applications.^8^^,^^9^

Patients enrolled in the study were informed that this was a health survey on quality of life, but the exact nature of the research question was not disclosed. Patients had to give consent to participate and to confirm their cancer diagnosis. The study population comprised cancer patients from 11 countries (Australia, Finland, France, Germany, Italy, Poland, Romania, Spain, Sweden, UK, and USA), representing various language groups and cultures. Participants were gender-balanced in each participating country.

### Statistics

#### Definition of equivalence margins

The starting point of our considerations regarding equivalence margins were limits that denote clinical importance based on empirical evidence and expert opinion.^10^^,^^11^ The minimal important difference (MID) is the smallest change in a disease outcome that a patient and/or a clinician would identify as relevant. The MIDs of the QLQ-C30 scales have been a matter of research and debate over the past 15 years. In a seminal publication, Osoba et al. proposed thresholds of 5 or 10 points for the PF, EF, SF, and QL scales.^12^ A more elaborate analysis based on a literature review and expert opinion was published by Cocks et al.^13^ Further publications by the EORTC QLG have defined MIDs, based on anchor variables, for each of the scales of the QLQ-C30 for various tumour sites.^14^, ^15^, ^16^, ^17^ A recent synthesis by Musoro et al. of 2023 summarized MIDs based on 21 clinical trials, showing that values vary from 4 to 15 points, depending on the type of cancer, the scale, and the direction of change (improvement or deterioration).^18^ Nevertheless, the vast majority of MIDs of the functional scales are clustered within a range of 5–10 points.

We employed a statistical advisory board to define an equivalence margin for the present study. After deliberation of the existing evidence, it was proposed to implement a conservative uniform equivalence threshold of <5 points for all functional scales under investigation.

#### Sample size

Sample size calculation was based on the primary objective of the study, which was to prove the equivalence of the QLQ-F17 and the functional part of the QLQ-C30. Therefore, the randomized independent group comparison of the two questionnaire versions was used as the basis for the calculation. A maximum mean difference of ±2 points between the QLQ-C30 and QLQ-F17 scales was conservatively assumed, as the scores were expected to be nearly identical given that both questionnaires included the same items. The standard deviation (SD) was also conservatively estimated at 30 for each scale, informed by the typical SD range (20–30) observed in the reference population. The equivalence margin was set to ]−5, 5[ for all scales. Under these assumptions, sample size was calculated to require a total of at least n = 2500 (i.e., n = 1250 per group) patients to reject the null hypothesis of non-equivalence at a 0·05 two-sided significance level with at least 80% power. Sample size calculation was performed using SAS v9·4 (SAS Institute, Cary, NC).

#### Methods of analyses

All statistical analyses were performed using the R Statistical Software (version 4.3.2; R Core Team 2024).^19^

The analysis population included all patients who participated in the survey and produced sufficient data quality. Poor data quality was an exclusion criterion and its definition is detailed in the Supplement.^20^

Patient characteristics are summarized for all patients and grouped by the respective order of the questionnaires (QLQ-C30—QLQ-F17 vs. QLQ-F17—QLQ-C30; Fig. 1). Continuous variables are presented using mean, SD, minimum, and maximum. Frequencies and percentages are presented for categorical variables.

#### Basic psychometric properties

Data from the first assessment of the QLQ-F17 (Fig. 1) were used for all basic psychometric analyses.

To investigate the 6 scales for the QLQ-F17 as analogous to the QLQ-C30, a 6-correlated factor model for confirmatory factor analysis (CFA) was performed. This was done using the lavaan package (v0·6–17). Standardized factor loadings and the comparative fit index (CFI), the Tucker–Lewis Index (TLI), as well as the root mean square error of approximation (RMSEA) are presented, and we refer to the publication of Kline to indicate an acceptable model fit.^21^

Reliability (internal consistency) of the scales of the QLQ-F17 was analysed using Cronbach's alpha coefficient. By convention, alpha values ≥0·70 are considered acceptable indicators for internal consistency.^22^ Construct validity was assessed by the item–scale correlations (multi-trait scaling analysis) using Pearsons correlation coefficient. A correlation between an item and its own scale >0·4 (corrected for overlap) was seen as evidence of the convergent validity of the item.^23^ Item-discriminant validity was assessed by comparing these correlations to the correlation coefficients of each item with other scales, considering that a definite scaling error exists if an item correlates less with its own scale than with another scale.^24^

#### Equivalence

Two main approaches were used to analyse the equivalence of the QLQ-C30 and the QLQ-F17. Between-group comparisons of the first QLQ assessment (QLQ-C30 first vs. QLQ-F17 first) and within-group comparisons (QLQ-C30 vs. QLQ-F17 within each patient, i.e., Assessment 1 vs. Assessment 2).

While no item order effects were expected for the first 7 items which captured PF and RF and were in the same position in both questionnaires, possible item order effects for the remaining 10 items measuring EF, CF, SF, and QL were of primary interest for all analyses. Hence, all differences detected for the first 7 items can be considered as random errors and were used as benchmarks for the remaining 10 items for the within-group and between-group analyses.

Between-group comparisons were done using Differential Item Functioning (DIF) and linear regression models. DIF assesses whether certain items within a test behave differently for different groups of individuals, even if those groups supposedly have similar levels of the construct being measured. Thus, DIF examines whether a single item functions differently between the QLQ-C30 and the QLQ-F17 while holding constant the underlying ability or trait being measured. To perform DIF detection, the lordif R package (v0·3–3; Seung W. Choi 2016) was used. The package applies ordinal logistic regression models, specifically cumulative logit models, in conjunction with trait-level scores derived from item response theory (IRT) as the matching criterion. The Brant test was used to assess whether the assumption of proportional odds held for each item. Uniform (constant effect) and non-uniform (effect varies conditional on the trait level) DIF was tested using a likelihood ratio (LR) χ^2^ test as the detection criterion at the level of significance of 0·01.^25^ Nagelkerke pseudo-R^2^ was used as the magnitude measure.^26^

Using multiple linear regression models, the mean score of each scale at the first assessment (QLQ-C30 at first assessment vs. QLQ-F17 at first assessment) was compared between the two questionnaires. The models were further adjusted for age, sex, country, current cancer status, Q168 (“To what extent have you been troubled with side-effects from your treatment?”), current treatment, and level of activity. These covariates were predefined by an expert panel and are also well-established predictors commonly used in similar analyses. Model assumptions - including normality of residuals, homogeneity of residual variance, linearity of continuous predictors, and absence of multicollinearity - were assessed using the R package “performance”. To account for potential heteroscedasticity near the scale boundaries (i.e., scores approaching 0 or 100), robust standard errors were used for the calculation of confidence intervals. Adjusted mean differences and 95%-confidence intervals are presented and were used for testing equivalence. Equivalence was shown if the upper and lower limits of the confidence intervals were within the equivalence margins ]−5, 5[.

The main method for the within-group comparisons to prove equivalence was linear mixed models. Linear mixed models with the factors “type of questionnaire” (QLQ-C30 or QLQ-F17), “period” (first or second assessment), the interaction of both factors and subject within sequence as random factor were calculated. Model assumptions–including normality of residuals, homogeneity of residual variance, and absence of multicollinearity–were assessed using the R package “performance”. The use of random intercepts was supported by high intraclass correlation coefficients (ICCs >0·7), indicating substantial between-patient variability; the random intercepts were assumed to follow a normal distribution with mean zero and an estimated variance component. Importantly, as shown by Schielzeth et al. (2020), slight violations of distributional assumptions in linear mixed-effects models typically do not lead to biased estimates, highlighting the robustness of the modeling approach.^27^ The adjusted mean difference between the two questionnaires was estimated together with the corresponding 95% confidence interval for the difference. Equivalence was assumed if the 95% confidence interval was fully within the equivalence interval of ]–5, 5[ points. This corresponds to an α = 5% test of equivalence.

Further supplemental analyses included item-level agreement, item-level test-retest reliability, and scale-level reliability. All supplemental analyses are based on within-group comparisons. To assess item-level agreement, for each item, the percentages of exact agreement were calculated as well as the percentages of differing by at most one response category (≤1 disagreement). Results of the first 7 items (same place and same order in both questionnaires) were compared to differences within items 8–17. Item-level test-retest reliability was analysed by using a weighted Kappa coefficient for all 17 items of the QLQ-F17. The intraclass–correlation coefficient (ICC) was used to assess scale-level reliability for each scale to investigate the relationship between measurement error and the variability between the subjects.

#### Role of the funding source

The study was funded by the EORTC QLG (Grant contract 01-2021). The funder reviewed and approved the study proposal but had no direct influence on the study design. The funder of the study was not involved in the collection, analysis, or interpretation of the data, nor in the drafting of the report. The final manuscript was submitted to and approved by the Executive Committee of EORTC QLG, and the manuscript is published on behalf of the EORTC QLG. Data are being shared according to the EORTC data sharing process. The EORTC QLG business model involves license fees for the commercial use of their instruments. Academic use of EORTC instruments is free of charge. The corresponding author had full access to all of the data and the final responsibility for the decision to submit the manuscript for publication.

## Results

### Dataset

**Fig. 2:**
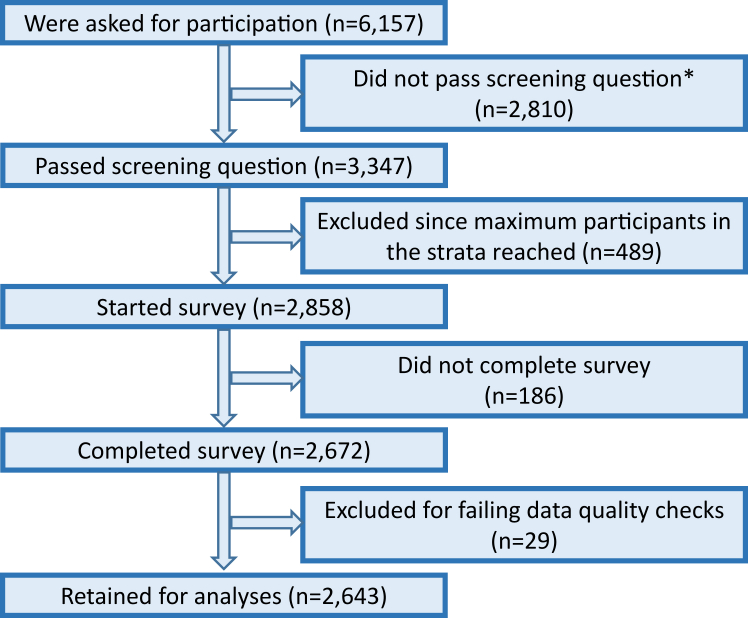
Participant flow. ∗Screening question: “Have you ever been diagnosed with cancer?”

A total of 2672 patients completed the survey between 17th February and 28th March 2023. After exclusion of n = 29 patients with poor data quality (see Supplementary Materials), the final sample size was n = 2643. Recruitment flow, survey completion numbers and exclusion due to data quality checks are shown in Fig. 2.

The final sample consisted of patients from 11 countries out of 6 language regions. Mean age was 58 years (range 18–92), and both sexes were equally distributed with 50% being male. Of all patients, 18% were currently undergoing cancer therapy, 74% were in remission from cancer, and 7% indicated that they had been newly diagnosed with cancer within the past 3 months. It is also comprised of all major cancer types with most cancer types being breast (21%), and prostate cancer (14%). All patient characteristics are presented in detail in Table 1.

**Table 1:** Patient characteristics (n = 2643).

	Overall (n = 2643)	QLQ-C30 first (n = 1320)	QLQ-F17 first (n = 1323)
**Sex, n (%)**			
Male	1311 (50%)	650 (49%)	661 (50%)
Female	1323 (50%)	664 (50%)	659 (50%)
I don't identify as either	7 (0·2%)	4 (0·3%)	3 (0·2%)
No answer given	2 (0·1%)	2 (0·1%)	0 (0%)
**Age, mean** ± **SD (min, max)**	58 ± 15 (18, 92)	58 ± 16 (18, 90)	58 ± 15 (18, 92)
**Cancer status, n (%)**			
I have been newly diagnosed with cancer within the past 3 months	190 (7%)	103 (8%)	87 (7%)
I am currently undergoing therapy for cancer	488 (18%)	257 (19%)	231 (17%)
I am in remission from cancer/I am a cancer survivor	1965 (74%)	960 (73%)	1005 (76%)
**To what extent have you been troubled with side-effects from your treatment (Q168)?, n (%)**			
Not at all	932 (35%)	480 (36%)	452 (34%)
A little	988 (37%)	493 (37%)	495 (37%)
Quite a bit	539 (20%)	269 (20%)	270 (20%)
Very much	184 (7%)	78 (6%)	106 (8%)
**Type of cancer, n (%)**			
Breast	564 (21%)	284 (22%)	280 (21%)
Prostate	373 (14%)	180 (14%)	193 (15%)
Skin	268 (10%)	126 (10%)	142 (11%)
Other gynaecological tumour (e.g., uterine, ovarian)	259 (10%)	125 (10%)	134 (10%)
Colorectal	208 (8%)	104 (8%)	104 (8%)
Lung	181 (7%)	103 (8%)	78 (6%)
Bladder	79 (3%)	46 (4%)	33 (3%)
Kidney	76 (3%)	32 (2%)	44 (3%)
Leukaemia	71 (3%)	35 (3%)	36 (3%)
Stomach	70 (3%)	33 (3%)	37 (3%)
Lymphoma	69 (3%)	36 (3%)	33 (3%)
Thyroid	68 (3%)	30 (2%)	38 (3%)
Oesophagus	64 (2%)	33 (3%)	31 (2%)
Testicular	41 (2%)	20 (2%)	21 (2%)
Liver	39 (2%)	21 (2%)	18 (1%)
Brain	30 (1%)	16 (1%)	14 (1%)
Myeloma	26 (1%)	13 (1%)	13 (1%)
Pancreas	17 (1%)	9 (1%)	8 (1%)
Other indication^a^	140 (5%)	74 (6%)	66 (5%)
**Time of cancer diagnosis, n (%)**			
Within 6 months	204 (8%)	104 (8%)	100 (8%)
Within 1 year	278 (11%)	141 (11%)	137 (10%)
Within 2 years	440 (17%)	220 (17%)	220 (17%)
Within 5 years	642 (24%)	319 (24%)	323 (24%)
Within 10 years	493 (19%)	243 (18%)	250 (19%)
More than 10 years ago	586 (22%)	293 (22%)	293 (22%)
**Type of active treatment (multiple answers possible), n (%)**			
Chemotherapy	291 (11%)	150 (11%)	141 (11%)
Immuno- or targeted therapy	283 (11%)	135 (10%)	148 (11%)
Radiotherapy	184 (7%)	89 (7%)	95 (7%)
Surgery within past 3 months	157 (6%)	81 (6%)	76 (6%)
**Level of activity, n (%)**			
Fully active, able to carry on all performances without restriction	607 (23%)	303 (23%)	304 (23%)
Active, but slightly restricted in physically strenuous activities	1020 (39%)	504 (38%)	516 (39%)
Limitations in activity and restricted in physically strenuous activities	698 (26%)	348 (26%)	350 (26%)
Capable of all self-care, but unable to carry out any work activities	249 (9%)	127 (10%)	122 (9%)
Capable of only limited self-care and confined to bed or chair more than 50% of waking hours	69 (3%)	38 (3%)	31 (2%)
**Highest completed level of education, n (%)**			
Below high school	367 (14%)	189 (14%)	178 (13%)
High school graduate	898 (34%)	461 (35%)	437 (33%)
College/Bachelor's degree/Master's degree	1115 (42%)	548 (42%)	567 (43%)
Doctorate	90 (3%)	40 (3%)	50 (4%)
Other	161 (6%)	76 (6%)	85 (6%)
Prefer not to answer	12 (0·5%)	6 (0·5%)	6 (0·5%)
**Country, n (%)**			
United States of America	420 (16%)	210 (16%)	210 (16%)
France	298 (11%)	149 (11%)	149 (11%)
Germany	297 (11%)	149 (11%)	148 (11%)
Italy	297 (11%)	148 (11%)	149 (11%)
Spain	297 (11%)	148 (11%)	149 (11%)
Poland	244 (9%)	121 (9%)	123 (9%)
United Kingdom	199 (8%)	99 (8%)	100 (8%)
Australia	150 (6%)	75 (6%)	75 (6%)
Finland	147 (6%)	75 (6%)	72 (5%)
Romania	147 (6%)	73 (6%)	74 (6%)
Sweden	147 (6%)	73 (6%)	74 (6%)
**Language regions, n (%)**			
Romance languages: French (France), Italian, Spanish (Spain), Romanian	1039 (39%)	518 (39%)	521 (39%)
English-speaking countries: UK, US, Australia	769 (29%)	384 (29%)	385 (29%)
West-Germanic language: German	297 (11%)	149 (11%)	148 (11%)
Slavic languages: Polish, Russian, Ukrainian	244 (9%)	121 (9%)	123 (9%)
Scandinavian language: Swedish	147 (6%)	73 (6%)	74 (6%)
Other European language: Finnish	147 (6%)	75 (6%)	72 (5%)

a Other were indicated as free text and include rare cancers like tongue cancer, squamous cell carcinoma, and gall bladder cancer or were not categorizable due to incomprehensible answers.

The median time for the total survey was 11:49 [IQR: 8:56–15:35] minutes. Median answering times for an item for each questionnaire, for the distractor questions, and for the whole survey are shown in Table 2.

**Table 2:** Time per item for each questionnaire and total time.

	10th percentile in mm:ss	Median in mm:ss	90th percentile in mm:ss
EORTC QLQ-C30			
Overall, total time (per item)	01:24 (00:03)	02:14 (00:04)	03:39 (00:07)
First assessment, total time (per item)	01:33 (00:03)	02:26 (00:05)	04:06 (00:08)
Second assessment, total time (per item)	01:19 (00:03)	02:03 (00:04)	03:15 (00:06)
EORTC QLQ-F17			
Overall, total time (per item)	00:49 (00:03)	01:24 (00:05)	02:23 (00:08)
First assessment, total time (per item)	00:59 (00:03)	01:36 (00:06)	02:44 (00:10)
Second assessment, total time (per item)	00:45 (00:03)	01:13 (00:04)	01:59 (00:07)
Time of distractor questions	04:21	07:36	14:54
Total time of survey	07:03	11:49	21:29

Time is shown as minutes:seconds (mm:ss).

Means and standard deviations of the functional scales derived from the QLQ-C30 in our study were practically identical to results reported in the EORTC reference manual, which is based on data from more than 23,000 cancer patients from numerous countries representing various age ranges, tumour sites as well as disease stages (Supplemental Table S1a).^28^ Furthermore, the symptom burden of our study sample is higher than that of the reference population (Supplemental Table S1b).

### Basic psychometric properties of the QLQ-F17 compared to the QLQ-C30

The CFA showed that factor loadings in the 6-factor model for the QLQ-F17 were all >0·4 with statistical significance. The model showed an acceptable fit (CFI = 0·941, TLI = 0·922, RMSEA = 0·079), and inter-factor correlations ranged from 0·44 to 0·84 (see Supplement Table S2).

The internal consistency of the scales of the QLQ-F17 was found to be in a very good range, with Cronbach's alpha coefficients between 0·73 and 0·89, almost identical to the coefficients of the QLQ-C30 (Table 3).

**Table 3:** Reliability and construct validity coefficients and descriptive statistics for EORTC QLQ-F17 and EORTC QLQ-C30 of the first assessment.

Scale	Questionnaire	Mean (SD)	Cronbach's alpha (95%-CI)	Corrected item–own scale correlation^a^	Item–other scale correlation	Scaling errors
Physical functioning	F17	75·6 (22·4)	0·84 (0·83, 0·86)	0·47–0·76	0·38–0·70	2 (8%)
	C30	75·7 (22·3)	0·84 (0·83, 0·86)	0·48–0·78	0·17–0·63	0 (0%)
Role functioning	F17	72·6 (28·6)	0·86 (0·85, 0·88)	0·76–0·76	0·41–0·68	0 (0%)
	C30	72·4 (28·8)	0·86 (0·85, 0·88)	0·76–0·76	0·46–0·72	0 (0%)
Emotional functioning	F17	65·1 (27·3)	0·89 (0·88, 0·90)	0·73–0·78	0·32–0·59	0 (0%)
	C30	67·8 (26·4)	0·89 (0·88, 0·90)	0·73–0·76	0·39–0·62	0 (0%)
Cognitive functioning	F17	77·3 (24·6)	0·73 (0·70, 0·76)	0·58–0·58	0·32–0·57	1 (10%)
	C30	75·8 (25·5)	0·72 (0·69, 0·75)	0·56–0·56	0·32–0·65	2 (20%)
Social functioning	F17	70·4 (29·8)	0·86 (0·84, 0·87)	0·75–0·75	0·44–0·66	0 (0%)
	C30	73·9 (29·1)	0·87 (0·85, 0·88)	0·77–0·77	0·46–0·69	0 (0%)
Global health status	F17	59·9 (21·2)	0·87 (0·85, 0·88)	0·77–0·77	0·19–0·51	0 (0%)
	C30	58·9 (21·7)	0·89 (0·87, 0·90)	0·79–0·79	0·37–0·50	0 (0%)

SD, standard deviation.

a Correlations of items with its own scale were calculated excluding that item for the total scale.

Multi-trait scaling analysis confirmed convergent validity for all items in the sense that items correlated substantially with their own scales (r > 0·4) of the QLQ-F17. Scaling errors (i.e., higher correlations of an item with another scale than with their own) were negligible, and these were primarily seen for the two CF items (Table 3). All item–scale correlations can be found in the Supplemental Table S3.

### Equivalence—between-group comparisons

#### Item level (DIF)

**Fig. 3:**
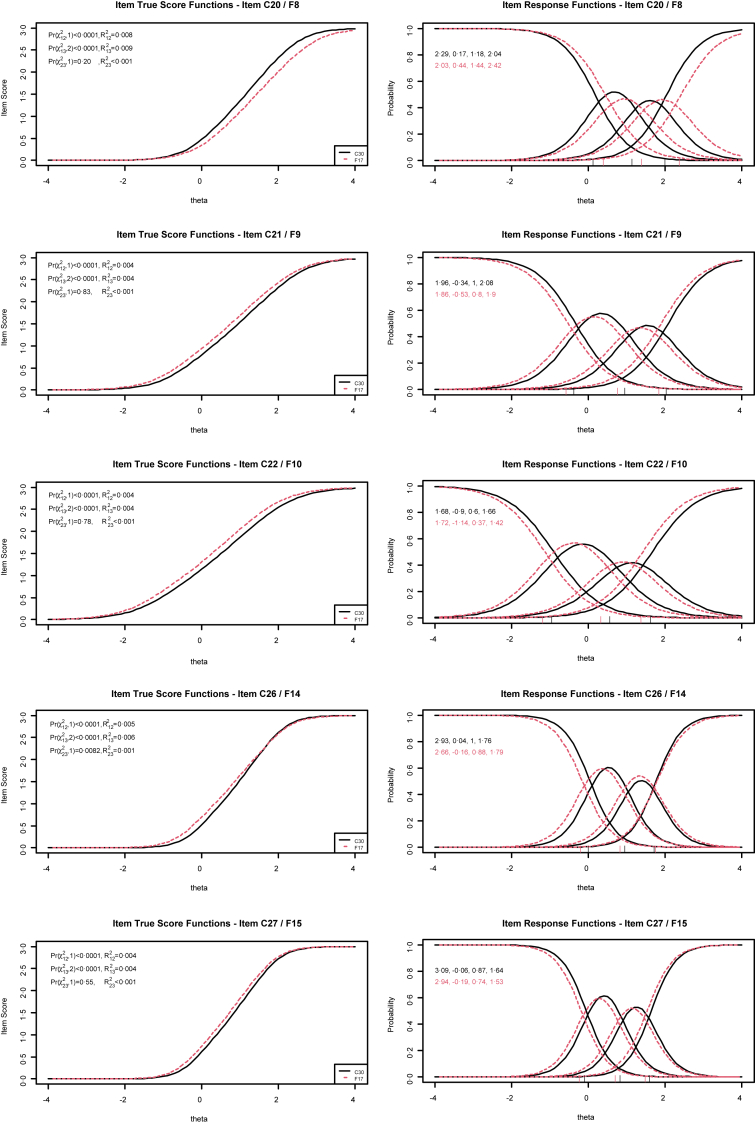
DIF of flagged items. Flagged items are defined by a p-value <0·01 of the likelihood ratio χ^2^ test to identify DIF (Table 4). The item true score functions on the left show the predicted item score in dependence of the trait level of the patients for both questionnaires. $\mathrm{Pr}\left(\chi_{12}^{2},1\right)$, p-value of the likelihood ratio χ^2^ test to identify uniform DIF; $\mathrm{Pr}\left(\chi_{13}^{2},1\right)$, p-value of the likelihood ratio χ^2^ test to test for an overall DIF; $\mathrm{Pr}\left(\chi_{23}^{2},1\right)$, p-value of the likelihood ratio χ^2^ test to identify non-uniform DIF; R^2^, effect size according to Nagelkerke. The item response functions on the right show the probability of the answering options in dependence of the trait level for both questionnaires. For example, a shift to the left of the QLQ-F17 compared to the QLQ-C30 (e.g., item C20/F8) indicates a higher item response for the QLQ-F17 compared to the QLQ-C30 under the same trait level. The numbers on the upper left represent slope and category threshold values by group.

Five items were identified as a type of questionnaire-related uniform DIF with question #8 (“Have you had difficulty in concentrating on things, like reading a newspaper or watching television?”), #9 (“Did you feel tense?”), #10 (“Did you worry?”), #14 (“Has your physical condition or medical treatment interfered with your family life?”), and #15 (“Has your physical condition or medical treatment interfered with your social activities?”), while a non-uniform DIF was seen only for question #14 (Table 4). A diagnostic plot is shown for each identified item in Fig. 3. The proportionate β_1_ change was very small for all 5 questions with a maximum change of 0·012 (i.e., about 1·2% change) for item #8, which is the item right after the Symptom items of the QLQ-C30. Nagelkerke's R^2^ values were consistently small, i.e., all R^2^ < 0·01. The direction of DIF differs for the scales. For question #8 (item of the CF scale) and #15 (item of the SF scale), the QLQ-F17 shows slightly lower scores compared to the QLQ-C30, whereas for questions #9 and #10 (both items of the EF scale) the opposite was the case, i.e., the QLQ-F17 showed higher scores. Question #14 (item of the SF scale) showed a non-uniform DIF with higher values for the QLQ-F17 for patients with a low trait and lower values for the QLQ-F17 for patients with a high trait (Fig. 3).

**Table 4:** Uniform and non-uniform differential item functioning statistics comparing common items of the EORTC QLQ-C30 and EORTC QLQ-F17.

Item #C30/F17 (scale)	Effect size uniform DIF^a^	p-value (uniform DIF)	Effect size non-uniform DIF^a^	p-value (non-uniform DIF)
1/1 (PF)	<0·001	0·60	<0·001	0·44
2/2 (PF)	<0·001	0·30	<0·001	0·37
3/3 (PF)	<0·001	0·38	<0·001	0·41
4/4 (PF)	<0·001	0·65	<0·001	0·93
5/5 (PF)	<0·001	0·66	0·002	0·026
6/6 (RF)	<0·001	0·93	<0·001	0·87
7/7 (RF)	<0·001	0·76	<0·001	0·49
20/8 (CF)	0·008	<0·0001	<0·001	0·20
21/9 (EF)	0·005	<0·0001	<0·001	0·83
22/10 (EF)	0·007	<0·0001	<0·001	0·78
23/11 (EF)	<0·001	0·50	<0·001	0·78
24/12 (EF)	<0·001	0·19	<0·001	0·58
25/13 (CF)	0·001	0·06	<0·001	0·74
26/14 (SF)	0·005	<0·0001	0·001	0·0082
27/15 (SF)	0·003	<0·0001	<0·001	0·55
29/16 (QL)	0·001	0·090	<0·001	0·80
30/17 (QL)	<0·001	0·45	<0·001	0·83

Results relate to the first assessment and compare respondents who either filled in the EORTC QLQ-C30 (n = 1320) or the EORTC QLQ-F17 (n = 1323). The first 7 items are on the same position in both questionnaires (block 1), the following items differ between QLQ-C30 and QLQ-F17 (block 2). Analyses are based on the between-group comparisons of phase 1. A p-value <0·01 indicates uniform or non-uniform differential item functioning. An effect size <0·01 can be considered as trivial and non-informative.

a Effect size according to Nagelkerke; PF, physical functioning; RF, role functioning; EF, emotional functioning; CF, cognitive functioning; SF, social functioning; QL, Global QoL/health status.

#### Multiple linear regression model

**Fig. 4:**
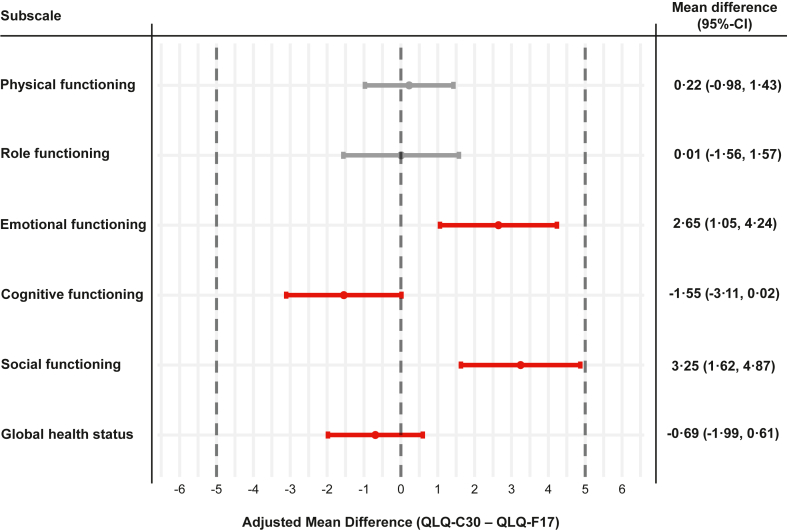
Forest plot of adjusted mean differences (QLQ-C30—QLQ-F17) of the first assessment period. The plot shows the adjusted mean differences of the multiple linear models comparing the scales of the QLQ-F17 and QLQ-C30 of the first assessment (phase 1). Linear models were adjusted for age, sex (“male”, “female”), country (“Australia”, “Finland”, “France”, ”Germany”, “Italy”, “Poland”, “Romania”, “Spain”, “Sweden”, “United Kingdom, “United States of America”), current cancer status (“I have been newly diagnosed with cancer within the past 3 months”, “I am currently undergoing therapy for cancer”, “I am in remission from cancer/I am a cancer survivor”), Q168 (“To what extent have you been troubled with side-effects from your treatment?”), current treatment (“Chemotherapy”, “Radiotherapy”, “Immuno—or targeted therapy”, “Surgery within past 3 months”, “Other therapy”, “No current treatment”) and level of activity (“Fully active, able to carry on all performance without restriction”, “Active, but slightly restricted in physically strenuous activities”, “Limitations in activity and restricted in physically strenuous activities”, “Capable of all self-care, but unable to carry out any work activities”, “Capable of only limited self-care and confined to bed or chair more than 50% of waking hours”). A total of n = 2683 patients were included in the models; 9 patients were excluded due to missing information on sex. The dotted vertical grey lines indicate the equivalence margin of ]−5, 5[. Grey scales consist of items on the same position in both questionnaires (block 1). Positions of items of red scales differ between QLQ-C30 and QLQ-F17 (block 2).

Multiple linear regression models were calculated for each scale. Nine patients (0·3%) were excluded from the analyses due to missing information on sex. The mean difference (QLQ-C30 – QLQ-F17) and the corresponding 95% confidence intervals of all scales lay within the predefined equivalence margin of ]−5, 5[. The results were in line with the DIF analyses, showing the highest difference between the two questionnaires for the EF and SF scales (Fig. 4).

### Equivalence—within-group comparisons

#### Linear mixed models

**Fig. 5:**
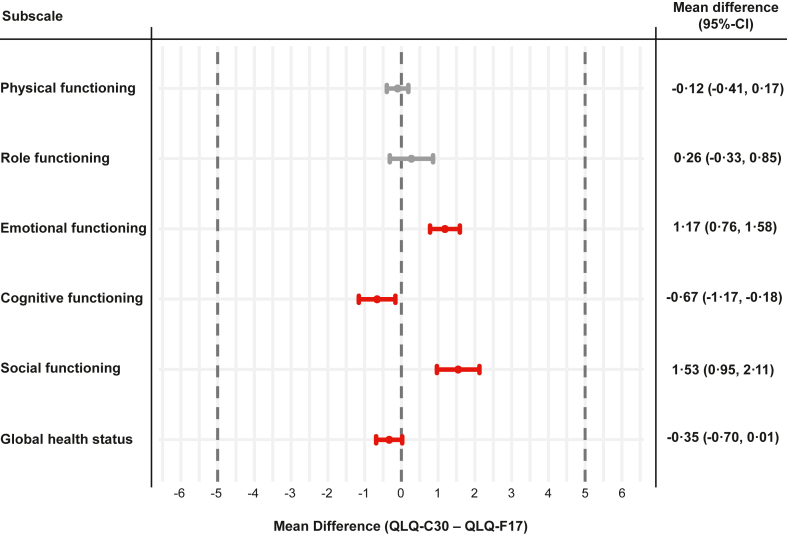
Forest plot of paired mean differences (QLQ-C30—QLQ-F17) between first and second assessment. The plot shows the mean difference of the linear mixed model comparing the scales of the QLQ-F17 and QLQ-C30 between Phase 1 and Phase 2 (cross-over design). The dotted vertical grey lines indicate the equivalence margin of ]−5, 5[. Grey scales consist of items on the same position in both questionnaires (block 1). Position of items of red scales differ between QLQ-C30 and QLQ-F17 (block 2).

**Fig. 6:**
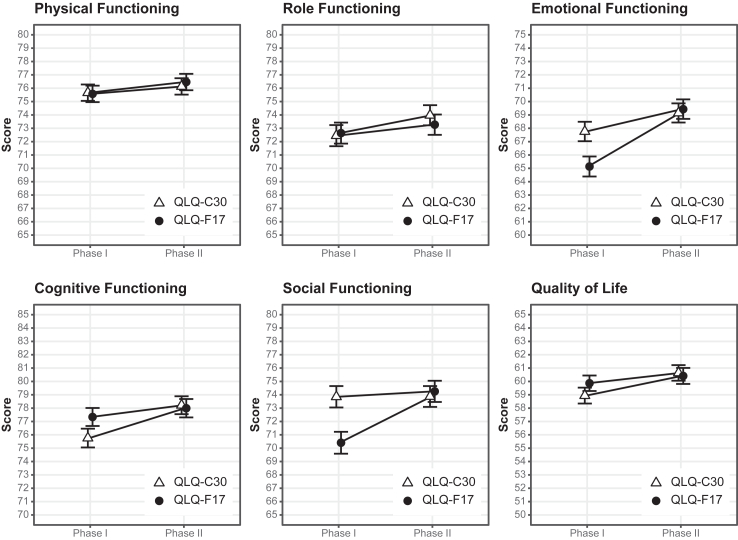
Mean differences between QLQ-F17 and QLQ-C30 depending on randomisation order and time of assessment. Data show mean values and corresponding 95%-confidence intervals for each scale in dependence on the time of assessment (Phase 1 and Phase 2) and the underlying type of questionnaire. The lines connect the QLQ-F17 and the QLQ-C30 depending on the randomization order.

The within-group analysis of equivalence showed mean differences close to 0 with narrow confidence intervals for all scales. The upper and lower limits of the 95%-CIs were all <5 points (equivalence margin), showing equivalence for all scales (Fig. 5). The interaction term treatment by period showed no significant effect for any of the scales. However, for EF, CF, and SF, a small difference between the QLQ-F17 and QLQ-C30 was observed for the first assessment while both questionnaires showed identical results in the second assessment indicating slight carryover effects (Fig. 6).^29^

#### Supporting/additional analyses

The percentage of exact agreement for each item (same answer in both questionnaires) ranged between 75% and 93% (median = 81%) for block 1 (i.e., first 7 items) and between 74% and 83% (median = 77%) for block 2 (i.e., items 10–17). The percentage of a maximum of one point of disagreement ranged between 98% and 99% (median = 98%) for block 1 and between 95% and 98% (median = 97%) for block 2 (Supplementary Table S4). Weighted Kappa coefficients were similar between all items, ranging between 0·62 and 0·75 for block 1 and between 0·63 and 0·75 for block 2 (Supplementary Table S4, Supplementary Figure S1). The ICCs were high for all scales (>0·8). The ICCs of the scales of block 2 lay between the ICCs of block 1 (Supplementary Figure S2).

## Discussion

This study employed a randomized cross-over design, whereby patients completed either QLQ-F17 or QLQ-C30 first, followed by the other version after a wash-out phase. The design allowed analysing equivalence as between (groups) as well as within (patient) comparisons. This was an international study and data were collected in 11 different countries thus ensuring cultural diversity.

The core finding was that both questionnaires under investigation showed equivalence for all functional scales and the Global Health Status/QL. As expected, measurement consistency was more pronounced in the within-group than in the between-group analyses.

Furthermore, basic psychometric properties (Cronbach's alpha, corrected item–own scale, and item–other scale correlations) of the QLQ-F17 matched those of the QLQ-C30. The CFA showed acceptable model fit estimates.

This study revealed that item order effects truly exist, in the sense that the order of items within a questionnaire influences responses to subsequent items. The PF and RF scales appear at the beginning of both questionnaires, and therefore yielded identical results for both questionnaire forms. The remaining scales (EF, CF, SF, and QL) are presented after several symptom items within the QLQ-C30; these symptom items were removed in the QLQ-F17. As expected, this subtle change resulted in pseudo-R^2^ values higher than zero. Though these DIF effects were statistically significant due to the large sample, effect sizes were negligibly small justifying our core conclusion: the two questionnaire versions are equivalent.^30^

The choice of the common equivalence margin of ]−5, 5[ for all scales was informed by a literature synthesis and approved by our statistical advisory board. In their comprehensive review, Musoro et al. summarised 21 clinical studies and presented MIDs for all QLQ-C30 scales across various cancer entities and study settings.^18^ These detailed results provide an excellent starting point when planning a clinical study for a given cancer type with the intent to choose a specific scale as the primary endpoint. At the same time, the synthesis makes clear that most MIDs are ≥5 and therefore lie outside the equivalence margin.

Our findings are based on a large, heterogeneous, cross-cultural sample of cancer patients, allowing for the generalizability of our results. The patient cohort consists of patients of all ages, from 11 different countries, and with different cancer diagnoses. Our descriptive findings on scale means and standard deviations for our entire sample (n = 2643) are practically identical for both the functional and the symptom scales to those reported in the EORTC reference values manual.^28^ It is somewhat surprising that our patients under the age of 50 report lower QoL scores than in the comparison data from the reference manual. This may be due to the fact that 42% of these patients were either newly diagnosed or under current therapy whereas this percentage was considerably lower in our other age groups (50–59: 24%, 60–69: 17%, ≥70: 18%).

A possible limitation of the study was the type of data acquisition. We commissioned a certified professional survey company, which maintains various patient panels, mainly for studies of the pharmaceutical industry. Thus, we had no direct contact with patients. Nevertheless, this kind of data collection is a common and valid method for collecting large patient samples within a limited time span (e.g., Nolte et al.^31^).

Another possible limitation relates to the time interval between the two measurements which were only divided by a 10-min wash-out phase. Other studies with test-retest assessments used intervals ranging from 3 h to 2 weeks; however, there is no consensus on which interval is preferable.^32^^,^^33^ A major problem with large intervals is that events may occur that render the second testing psychologically dissimilar from the first testing; hence, test-retest consistency cannot be expected. On the other hand, a test re-administered too soon after the first one could allow for a recall of memorized answers. Our data, however, showed that despite the short time interval, responses to the same items were not identical but showed some variation around the true score. Implementing a separate second assessment point in the context of a survey was not an option due to high costs and the considerable risk of losing patients for the second assessment point (attrition bias). In addition, we point out that the first assessment was based on a randomised design, allowing for the most rigorous comparison method according to current scientific standards.

The QLQ-F17, serving as a core questionnaire for evaluating patient functioning analogous to the QLQ-C30, can be supplemented with additional questions selected from the EORTC Item Library.^34^ These supplementary items may be chosen to address disease-related symptoms and adverse events anticipated in the context of the treatment under investigation. Such a strategy allows for more flexibility in PRO assessment, staying in line with the FDA recommendation published in 2024 for assessing core patient-reported outcomes while minimizing patient burden.^35^ This strategy also echoes users’ frequently expressed need for a shorter and validated version of the QLQ-C30.

In conclusion, the QLQ-F17 is a reliable instrument for assessing cancer patients' self-reported functioning and yields measurement results that are equivalent to the functional part of the QLQ-C30. Using the generic QLQ-F17 as a core questionnaire along with symptom items from the EORTC QLG Item Library allows for a time-economic and flexible testing strategy in cancer clinical trials and practice.

## Contributors

MK and FZ designed the study, were responsible for the conduct of the study, drafted the first version of the paper, and approved the final manuscript. FZ was responsible for the statistical analyses. CC, KC, JG, MG, GI, DK, LL, SN, CP, MAP, TP, and SS helped in designing the study and/or interpreting the study results and/or commented on previous versions of the manuscript. All authors approved the final manuscript.

## Data sharing statement

Data will be shared according to the EORTC data release policy (https://www.eortc.org/data-sharing/).

## Declaration of interests

All authors have completed the ICMJE uniform disclosure form at https://www.icmje.org/disclosure-of-interest/:

Florian Zeman: Study was funded by the European Organisation for Research and Treatment of Cancer (EORTC) Quality of Life Group; received support by the EORTC for attending meetings and/or travel for the biannual QLG Meetings.

Johannes Maria Giesinger: none.

Tobias Pukrop: none.

Morten Aagaard Petersen: none.

Mogens Groenvold: none.

Sandra Nolte: none.

Dagmara Kuliś: none.

Sunil Shrestha: none.

Laurence Leysen: none.

Kim Cocks: none.

Corneel Coens: full-time employee at the EORTC.

Georgios Ioannidis: none.

Cecilia Pompili: recieved consulting fees and support for attending meetings and/or travel from AZ, J&J, Medela, BD; received payment or honoraria for lectures, presentations, speakers bureaus, manuscript writing or educational events from AZ; participation on a Data Safety Monitoring Board or Advisory Board from AZ.

Michael Koller: Study was funded by the European Organisation for Research and Treatment of Cancer (EORTC) Quality of Life Group; received support by the EORTC for attending meetings and/or travel for the biannual QLG Meetings.

## Appendix A Supplementary data

Figure S1 Weighted Kappa for each item comparing the QLQ-F17 and QLQ-C30. Grey bars present items on the same position in both questionnaires (block 1). Position of items of red bars differ between QLQ-C30 and QLQ-F17 (block 2).

Figure S2 Intraclass correlation coefficient (ICC) of each scale comparing the QLQ-C30 and QLQ-F17. Error bars present 95%-confidence intervals. Grey scales consist of items on the same position in both questionnaires (block 1). Position of items of red scales differ between QLQ-C30 and QLQ-F17 (block 2).

Supplemental Tables

Supplement Data Quality
