# Supplementary figures and images for: The EORTC QLQ-F17 as a shortened version of the EORTC QLQ-C30 to assess self-reported functioning in cancer patients: investigating equivalence and psychometric properties in a randomized cross-over trial
Source: eClinicalMedicine. 2025 Jun 3;84:103262. doi: 10.1016/j.eclinm.2025.103262 (PMC12167450; doi:10.1016/j.eclinm.2025.103262)

## Slide 1
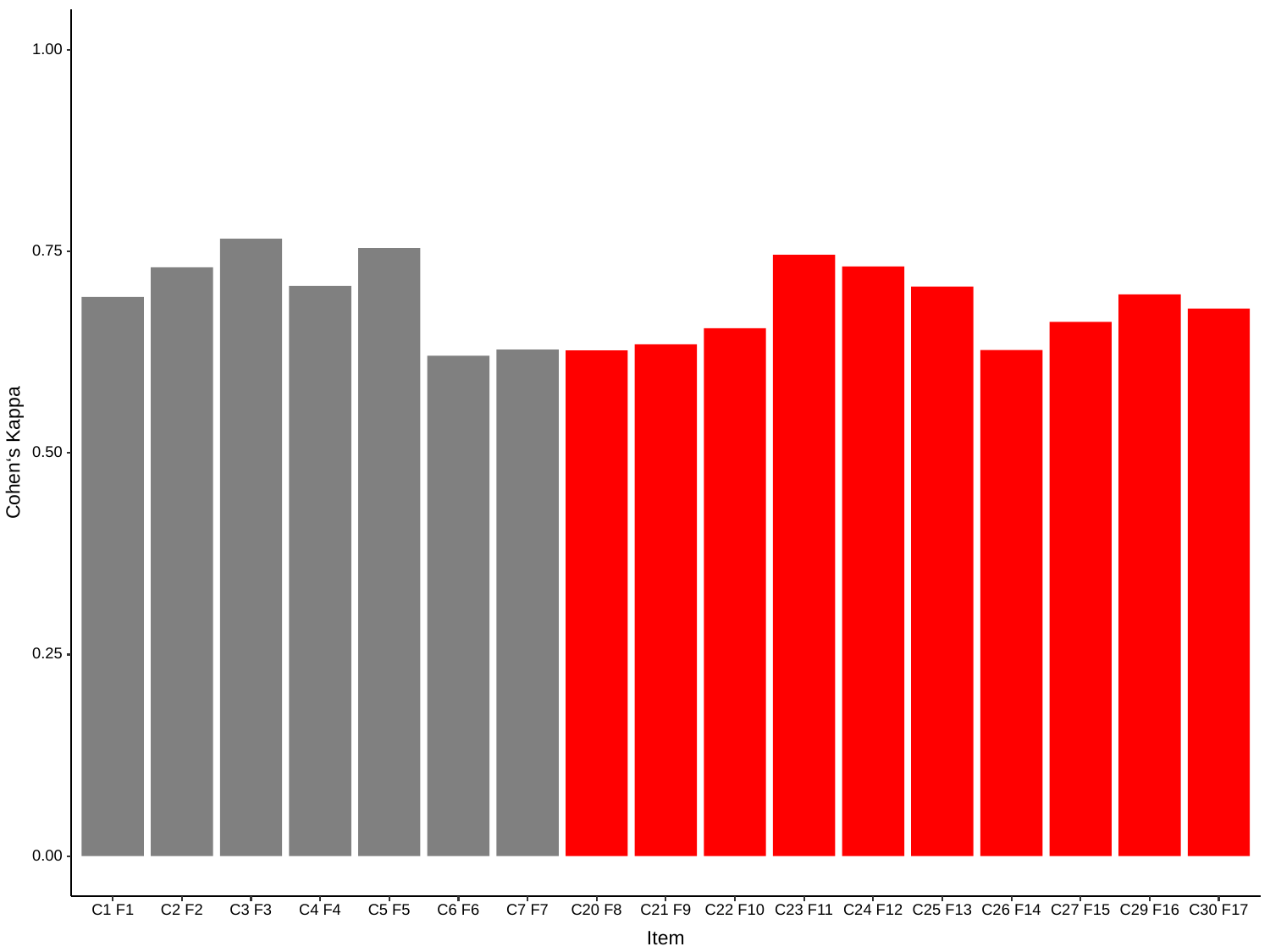

1.00
0.75
Cohen‘s Kappa
0.50
0.25
0.00
C3 F3
C23 F11
C25 F13
C30 F17
C1 F1
C2 F2
C4 F4
C5 F5
C6 F6
C7 F7
C20 F8
C21 F9
C22 F10
C24 F12
C26 F14
C27 F15
C29 F16
Item

Supplement: Figure S1 — Weighted Kappa for each item comparing the QLQ-F17 and QLQ-C30. Grey bars present items on the same position in both questionnaires (block 1). Position of items of red bars differ between QLQ-C30 and QLQ-F17 (block 2). [file mmc1.pptx]

## Slide 1
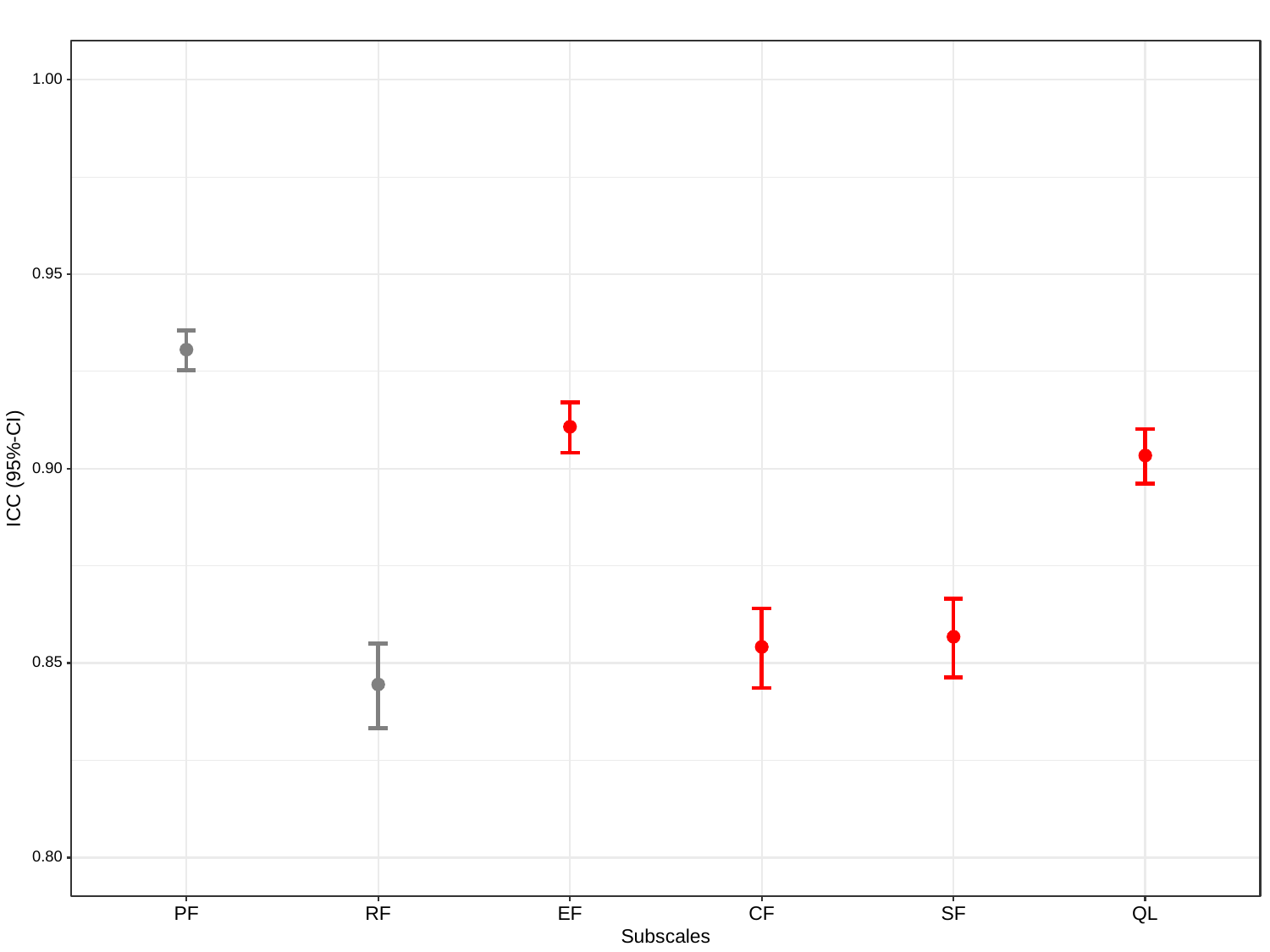

1.00
0.95
ICC (95%-CI)
0.90
0.85
0.80
QL
CF
SF
PF
RF
EF
Subscales

Supplement: Figure S2 — Intraclass correlation coefficient (ICC) of each scale comparing the QLQ-C30 and QLQ-F17. Error bars present 95%-confidence intervals. Grey scales consist of items on the same position in both questionnaires (block 1). Position of items of red scales differ between QLQ-C30 and QLQ-F17 (block 2). [file mmc2.pptx]
