# Supplementary material for: The EORTC QLQ-F17 as a shortened version of the EORTC QLQ-C30 to assess self-reported functioning in cancer patients: investigating equivalence and psychometric properties in a randomized cross-over trial
Source: eClinicalMedicine. 2025 Jun 3;84:103262. doi: 10.1016/j.eclinm.2025.103262 (PMC12167450; doi:10.1016/j.eclinm.2025.103262)
Supplement: Supplemental Tables [file mmc3.docx]

**Table S1a**: EORTC QLQ-C30 mean values of functional scales compared to reference values by age and in total

| Age-group | Population | PF | RF | EF | CF | SF | QL |
| --- | --- | --- | --- | --- | --- | --- | --- |
| <50 | Study group (n=739)* | 68·6  (SD 25·0) | 63·8  (SD 29·2) | 53·1  (SD 27·4) | 64·0  (SD 29·0) | 61·3  (SD 30·7) | 57·5  (SD 22·2) |
|  | Reference population (n=5,207)** | 80·2  (SD 20·8) | 68·6  (SD 31·7) | 69·2  (SD 24·4) | 82·9  (SD 21·6) | 72·1  (SD 29·5) | 61·4  (SD 23·4) |
| 50-59 | Study group (n=484)* | 78·3  (SD 21·6) | 72·0  (SD 28·7) | 64·8  (SD 26·2) | 75·5  (SD 25·4) | 72·6  (SD 29·2) | 58·4  (SD 21·8) |
|  | Reference population (n=5,707)** | 78·0  (SD 22·5) | 69·4  (SD 32·7) | 69·0  (SD 24·2) | 83·2  (SD 21·9) | 73·5  (SD 29·4) | 61·2  (SD 24·1) |
| 60-69 | Study group (n=730)* | 78·9  (SD 20·1) | 77·2  (SD 26·5) | 74·2  (SD 22·9) | 83·4  (SD 20·4) | 79·3  (SD 26·0) | 60·5  (SD 21·1) |
|  | Reference population (n=6,709)** | 76·3  (SD 23·5) | 72·6  (SD 32·7) | 71·8  (SD 24·3) | 83·1  (SD 21·6) | 76·4  (SD 28·8) | 61·8  (SD 24·4) |
| ≥70 | Study group (n=690)* | 78·8  (SD 20·3) | 79·9  (SD 25·2) | 81·3  (SD 18·6) | 85·1  (SD 17·6) | 82·5  (SD 23·8) | 62·5  (SD 20·6) |
|  | Reference population (n=5,357)** | 72·1  (SD 25·4) | 70·7  (SD 34·1) | 76·1  (SD 23·2) | 81·0  (SD 22·4) | 78·2  (SD 28·2) | 60·6  (SD 25·1) |
| total | Study group (n=2,643)* | 75·9  (SD 22·3) | 73·2  (SD 28·1) | 68·5  (SD 26·3) | 77·0  (SD 25·0) | 73·9  (SD 28·7) | 59·8  (SD 21·5) |
|  | Reference population (n=23,553)** | 76·7  (SD 23·2) | 70·5  (SD 32·8) | 71·4  (SD 24·2) | 82·6  (SD 21·9) | 75·0  (SD 29·1) | 61·3  (SD 24·2) |

PF, physical functioning; RF, role functioning; EF, emotional functioning; CF, cognitive functioning; SF, social functioning; QL, Global Quality of Life (QOL)/health status; * percentage of patients newly diagnosed or under current therapy within each age-group: 42% (<50y), 24% (50-59y), 17% (60-69y), 18% (≥70y). **Reference population is mainly based on data from cancer clinical trials and epidemiological studies with 23,553 cases according to Scott et al.^28^

**Table S1b**: EORTC QLQ-C30 mean values of symptom scales compared to reference values by cancer status and in total

| Population | FA | NV | PA | DY | SL | AP | CO | DI |
| --- | --- | --- | --- | --- | --- | --- | --- | --- |
| Subgroups by Cancer Status |  |  |  |  |  |  |  |  |
| Newly diagnosed with cancer within the past 3 months (n=190) | 51·5  (SD 30·3) | 34·4  (SD 35·0) | 46·8  (SD 34·0) | 40·0  (SD 34·5) | 50·7  (SD 35·6) | 41·2  (SD 37·3) | 37·7  (SD 36·1) | 28·8  (SD 34·8) |
| Currently undergoing therapy for cancer (n=488) | 48·8  (SD 26·9) | 20·6  (SD 27·1) | 40·2  (SD 29·2) | 34·6  (SD 29·9) | 44·0  (SD 32·3) | 27·4  (SD 30·5) | 27·5  (SD 30·6) | 19·7  (SD 27·1) |
| Remission from cancer / cancer survivor (n=1,965) | 34·7  (SD 25·0) | 7·7  (SD 16·8) | 30·2  (SD 27·8) | 21·4  (SD 27·6) | 34·6  (SD 30·8) | 12·9  (SD 23·5) | 15·4  (SD 24·0) | 12·0  (SD 22·3) |
| Study group (n=2,643) | 38·5  (SD 26·6) | 12·0  (SD 22·3) | 33·2  (SD 29·0) | 25·2  (SD 29·3) | 37·5  (SD 31·8) | 17·6  (SD 27·5) | 19·2  (SD 27·3) | 14·6  (SD 24·8) |
| Reference population (n=23,553)* | 34·6  (SD 27·8) | 9·1  (SD 19·0) | 27·0  (SD 29·9) | 21·0  (SD 28·4) | 28·9  (SD 31·9) | 21·1  (SD 31·3) | 17·5  (SD 28·4) | 9·0  (SD 20·3) |

FA, Fatigue; NV, Nausea and vomiting; PA, Pain; DY, Dyspnoea; SL, Insomnia; AP, Appetite loss; CO, Constipation; DI, Diarrhoea; *Reference population is mainly based on data from cancer clinical trials and epidemiological studies with 23,553 cases according to Scott et al.^28^

**Table S2**: Confirmatory factor analysis of the EORTC QLQ-F17 (N=1323, first assessment) showing standardized factor loadings and scaled fit indices

| **Item** | **PF** | **RF** | **EF** | **CF** | **SF** | **QL** |
| --- | --- | --- | --- | --- | --- | --- |
| F1 | 0·781 | 0 | 0 | 0 | 0 | 0 |
| F2 | 0·796 | 0 | 0 | 0 | 0 | 0 |
| F3 | 0·810 | 0 | 0 | 0 | 0 | 0 |
| F4 | 0·712 | 0 | 0 | 0 | 0 | 0 |
| F5 | 0·535 | 0 | 0 | 0 | 0 | 0 |
| F6 | 0 | 0·880 | 0 | 0 | 0 | 0 |
| F7 | 0 | 0·866 | 0 | 0 | 0 | 0 |
| F9 | 0 | 0 | 0·829 | 0 | 0 | 0 |
| F10 | 0 | 0 | 0·829 | 0 | 0 | 0 |
| F11 | 0 | 0 | 0·784 | 0 | 0 | 0 |
| F12 | 0 | 0 | 0·833 | 0 | 0 | 0 |
| F8 | 0 | 0 | 0 | 0·802 | 0 | 0 |
| F13 | 0 | 0 | 0 | 0·725 | 0 | 0 |
| F14 | 0 | 0 | 0 | 0 | 0·845 | 0 |
| F15 | 0 | 0 | 0 | 0 | 0·890 | 0 |
| F16 | 0 | 0 | 0 | 0 | 0 | 0·865 |
| F17 | 0 | 0 | 0 | 0 | 0 | 0·885 |
| 6-Factor Model fit estimates of the QLQ-F17:  CFI=0·941, TLI=0·922, RMSEA=0·079 (90%-CI: 0·075; 0·084) | | | | | | |

PF, physical functioning; RF, role functioning; EF, emotional functioning; CF, cognitive functioning; SF, social functioning; QL, Global QOL/health status; CFI, comparative fit index; TLI, Tucker-Lewis Index; RMSEA, root mean square error of approximation

**Table S3**: Corrected item-scale correlations of QLQ-F17

| **Scale** | **Item** | **PF** | **RF** | **EF** | **CF** | **SF** | **QL** |
| --- | --- | --- | --- | --- | --- | --- | --- |
| PF | F1 | **0·69** | 0·62 | 0·39 | 0·41 | 0·53 | 0·47 |
| PF | F2 | **0·71** | 0·58 | 0·32 | 0·35 | 0·50 | 0·47 |
| PF | F3 | **0·76** | 0·61 | 0·35 | 0·40 | 0·51 | 0·37 |
| PF | F4 | **0·64** | 0·59 | 0·44 | 0·45 | 0·58 | 0·41 |
| PF | F5 | **0·47** | 0·50 | 0·39 | 0·49 | 0·44 | 0·19 |
| RF | F6 | 0·70 | **0·76** | 0·49 | 0·51 | 0·66 | 0·51 |
| RF | F7 | 0·68 | **0·76** | 0·51 | 0·51 | 0·65 | 0·47 |
| EF | F9 | 0·44 | 0·50 | **0·77** | 0·56 | 0·53 | 0·39 |
| EF | F10 | 0·38 | 0·47 | **0·78** | 0·50 | 0·54 | 0·40 |
| EF | F11 | 0·41 | 0·41 | **0·73** | 0·54 | 0·52 | 0·37 |
| EF | F12 | 0·41 | 0·47 | **0·76** | 0·57 | 0·56 | 0·47 |
| CF | F8 | 0·48 | 0·52 | 0·59 | **0·58** | 0·54 | 0·32 |
| CF | F13 | 0·45 | 0·45 | 0·53 | **0·58** | 0·50 | 0·31 |
| SF | F14 | 0·59 | 0·63 | 0·58 | 0·55 | **0·75** | 0·46 |
| SF | F15 | 0·63 | 0·68 | 0·58 | 0·55 | **0·75** | 0·49 |
| QL | F16 | 0·49 | 0·50 | 0·40 | 0·32 | 0·47 | **0·77** |
| QL | F17 | 0·46 | 0·48 | 0·48 | 0·34 | 0·48 | **0·77** |

PF, physical functioning; RF, role functioning; EF, emotional functioning; CF, cognitive functioning; SF, social functioning; QL, Global QOL/health status;

**Table S4**: Item-level agreement and weighted Kappa (item-level test-retest reliability)

|  | Absolute agreement (95%-CI) | ≤1 disagreement*  (95%-CI) | Weighted Kappa  (95%-CI) |
| --- | --- | --- | --- |
| 1/1 (PF) | 78.7% (77.1%, 80.3%) | 98.5% (97.9%, 98.9%) | 0.72 (0.69, 0.74) |
| 2/2 (PF) | 80.8% (79.2%, 82.3%) | 98.9% (98.4%, 99.3%) | 0.75 (0.72, 0.78) |
| 3/3 (PF) | 87.6% (86.3%, 88.9%) | 98.7% (98.2%, 99.1%) | 0.66 (0.62, 0.71) |
| 4/4 (PF) | 82.7% (81.2%, 84.1%) | 98.3% (97.8%, 98.8%) | 0.64 (0.60, 0.68) |
| 5/5 (PF) | 93.5% (92.4%, 94.4%) | 98.9% (98.4%, 99.2%) | 0.60 (0.53, 0.68) |
| 6/6 (RF) | 75.3% (73.6%, 76.9%) | 98.0% (97.4%, 98.5%) | 0.59 (0.55, 0.62) |
| 7/7 (RF) | 75.8% (74.1%, 77.4%) | 97.7% (97.1%, 98.3%) | 0.61 (0.58, 0.65) |
| 20/8 (CF) | 79.0% (77.4%, 80.5%) | 97.2% (96.5%, 97.8%) | 0.54 (0.49, 0.59) |
| 21/9 (EF) | 75.5% (73.8%, 77.1%) | 97.8% (97.2%, 98.4%) | 0.61 (0.57, 0.64) |
| 22/10 (EF) | 75.6% (73.9%, 77.2%) | 96.9% (96.2%, 97.5%) | 0.71 (0.68, 0.74) |
| 23/11 (EF) | 83.2% (81.8%, 84.6%) | 98.9% (98.5%, 99.3%) | 0.75 (0.72, 0.78) |
| 24/12 (EF) | 82.0% (80.4%, 83.4%) | 98.1% (97.5%, 98.6%) | 0.72 (0.69, 0.75) |
| 25/13 (CF) | 81.3% (79.8%, 82.8%) | 98.3% (97.8%, 98.8%) | 0.67 (0.63, 0.71) |
| 26/14 (SF) | 76.2% (74.5%, 77.8%) | 97.1% (96.4%, 97.7%) | 0.57 (0.53, 0.61) |
| 27/15 (SF) | 77.6% (76.0%, 79.2%) | 97.4% (96.7%, 98.0%) | 0.64 (0.61, 0.68) |
| 29/16 (QoL) | 75.9% (74.3%, 77.6%) | 95.0% (94.1%, 95.8%) | 0.72 (0.69, 0.74) |
| 30/17 (QoL) | 74.5% (72.8%, 76.1%) | 95.3% (94.5%, 96.1%) | 0.75 (0.72, 0.78) |

* differing by at most one response category
