# Supplementary material for: The EORTC QLQ-F17 as a shortened version of the EORTC QLQ-C30 to assess self-reported functioning in cancer patients: investigating equivalence and psychometric properties in a randomized cross-over trial
Source: eClinicalMedicine. 2025 Jun 3;84:103262. doi: 10.1016/j.eclinm.2025.103262 (PMC12167450; doi:10.1016/j.eclinm.2025.103262)
Supplement: Supplement Data Quality [file mmc4.docx]

**Data Quality Check**

To ensure good quality data, the following checks were used to identify careless responders:

1) Consistency check of the first 7 items of the QLQ-C30 and the QLQ-F17

The first 7 items of the QLQ-C30 and the QLQ-F17 are identical; hence, no item order effects are expected. All items are rated on a 4-point scale (‘Not at all’; ‘a little’; ‘quite a bit’; ‘very much’). Inconsistency was defined if the response to an item was the exact opposite between both questionnaires (‘not at all’ versus ‘very much’). Respondents with opposite answers were flagged (Flag #1; Table A1)

2) Response time of each questionnaire as well as for the distractor questions was recorded and analyzed. All fast responders were flagged (Table A2), while a fast responder was defined as:

- Total interview time faster than half of the median of all participants (Flag #2)
- Average time for an item of the QLQ-Q30 or QLQ-F17 faster than 2 seconds (Flag #3)

**Table A1:** Data quality check of opposite response patterns

| **Item QLQ-C30/ QLQ-F17** | **Answering pattern**  **QLQ-C30 vs QLQ-F17** | **Number of flagged participants** |
| --- | --- | --- |
| #1: Do you have any trouble doing strenuous activities, like carrying a heavy shopping bag or a suitcase? | Very much vs. not at all  Not at all vs. very much | 5 (0·2%)  2 (0·1%) |
| #2: Do you have any trouble taking a long walk? | Very much vs. not at all  Not at all vs. very much | 4 (0·2%)  0 (0·0%) |
| #3: Do you have any trouble taking a short walk outside of the house? | Very much vs. not at all  Not at all vs. very much | 5 (0·2%)  4 (0·2%) |
| #4: Do you need to stay in bed or a chair during the day? | Very much vs. not at all  Not at all vs. very much | 4 (0·2%)  0 (0·0%) |
| #5: Do you need help with eating, dressing, washing yourself or using the toilet? | Very much vs. not at all  Not at all vs. very much | 5 (0·2%)  2 (0·1%) |
| #6: Were you limited in doing either your work or other daily activities? | Very much vs. not at all  Not at all vs. very much | 3 (0·1%)  10 (0·4%) |
| #7: Were you limited in pursuing your hobbies or other leisure time activities? | Very much vs. not at all  Not at all vs. very much | 8 (0·3%)  5 (0·2%) |

**Table A2:** Data quality check of response times

| Time measurement | Cut-off | Number of flagged participants |
| --- | --- | --- |
| Total interview time | <5.89 minutes* | 146 (5·5%) |
| Average answering time of C30 | <2secs | 69 (2·6%) |
| Average answering time of F17 | <2secs | 62 (2·3%) |

* half of median time of all participants

Participants with 3 or more flags were excluded from the analysis set due to bad data quality (Table A3).

**Table A3:** Definition of analysis population according to data quality checks

| Total number of flags | Frequency (%) | Decision |
| --- | --- | --- |
| 0 | 2469 (92·4%) | Analysis population |
| 1 | 113 (4·2%) |  |
| 2 | 61 (2·3%) |  |
| 3 | 22 (0·8%) | Excluded from the dataset |
| 4 | 4 (0·2%) |  |
| 5 | 2 (0·1%) |  |
| 6 | 0 (0·0%) |  |
| 7 | 1 (0·04%) |  |
